# Technique and clinical results of a new intramedullary support nail and plate system for fixation of 3- or 4- part proximal humeral fractures in older adults

**DOI:** 10.1186/s12891-022-05998-z

**Published:** 2022-11-30

**Authors:** Xuedong Bai, Zhengguo Zhu, Zuhao Chang, Lijun Sun, Peifu Tang, Hua Chen

**Affiliations:** 1 Department of Orthopedics, Chinese PLA General Hospital, Haidian District, No. 28, Fuxing Road, Beijing, 100853 China; 2 National Clinical Research Center for Orthopedics, Sports Medicine and Rehabilitation, No. 28, Fuxing Road, Haidian District, Beijing, 100853 China

**Keywords:** Proximal humeral fracture, Support, Nail, Locking plate, Osteoporosis

## Abstract

**Background:**

Internal fixation of complex proximal humeral fractures (PHF) with osteoporosis is associated with a high incidence of complications. This study introduces the technique and clinical results of a novel intramedullary support nail and plate system (ISNPs) for the internal fixation of 3- or 4- part PHF in older adults. The ISNPs combines the concept of intramedullary support and dynamic fixation into a locking plate fixation system that can be applied using a minimally invasive surgical approach.

**Methods:**

A total of 46 consecutive patients diagnosed with 3- or 4-part PHF that met the criteria were included in this study, including 18 in the ISNPs group and 28 in the conventional locking plate (LP) group. Clinical results, including operative time, intraoperative bleeding, reduction quality, subjective outcome ratings, and complications, were compared between the two groups. Functional outcomes were evaluated using the Constant score and disability of the arm, shoulder, and hand (DASH) questionnaire at 1-year follow-up.

**Results:**

There were no significant differences in age, sex, local bone quality, Neer-fracture type, and follow-up time between the ISNPs and LP groups. For clinical analysis, there were no significant differences in intraoperative bleeding and operation time between the ISNPs and LP groups. Significant differences were observed in the percentage of the malreduced cases, Constant and DASH score analysis, and the patients’ subjective evaluation (‘excellent’ and ‘good’ %) between the two groups.

**Conclusion:**

The ISNPs technique proposed in this study provides a novel hybrid internal fixation model for complex PHF with osteoporosis. The clinical results at 1-year follow-up confirmed the advantage of applying it to 3- or 4- part PHF in older patients. Further studies are required to optimize its design and explore its optimal indications.

**Supplementary Information:**

The online version contains supplementary material available at 10.1186/s12891-022-05998-z.

## Introduction

With the aging of the world population, the incidence of proximal humeral fractures (PHF) continues to rise, which has become a major public health concern in many countries [1, 2]. Many PHF cases can be managed nonsurgically with acceptable outcomes. However, for fractures with significant displacement and tuberosity malalignment, surgery is often considered necessary [3, 4]. Although arthroplasty has become a common treatment for 3- or 4-part fractures in many clinics, and achieved good clinical results, internal fixation with locking plates and intramedullary nails as the main implants remains the most commonly used method for acute PHF treatment [1, 5, 6]. However, throughout the literature, internal fixation of 3- or 4-part PHF has been associated with considerable complication rates, particularly in the presence of osteoporosis [2, 7–9]. Therefore, new internal fixation techniques are required to treat complex PHF with osteoporosis.

Previously, our studies found that locking plate fixation with fibular strut allograft augmentation potentially achieves superior clinical results to those of locking plate fixation alone in treating 3- or 4-part PHF with osteoporosis [10, 11], a phenomenon that has also been confirmed by several other reports [12–15]. Subsequently, we developed an anatomical fibular strut allograft, which was shaped according to the medullary cavity’s geometry, and achieved superior biomechanical and clinical results [11, 16]. However, the source limitations and inherent drawbacks of allograft transplantation limit its clinical application. The success of intramedullary fibular allografts may be attributed to its intramedullary mechanical support and biomaterial properties. We believe that early intramedullary mechanical support of the humeral head is the main mechanism to promote fracture healing. Therefore, we further studied the anatomical characteristics of the proximal humerus in older adults [17], and developed an intramedullary support nail and plate system (ISNPs). To the best of our knowledge, this is the first attempt to combine the advantages of locking plates, intramedullary support, and dynamic screw fixation into a hybrid fixation system. Herein, we describe the surgical technique and 1-year clinical results of the ISNPs and compare them with those associated with conventional locking plates.

## Materials and methods

### The ISNPs design

**Fig. 1:**
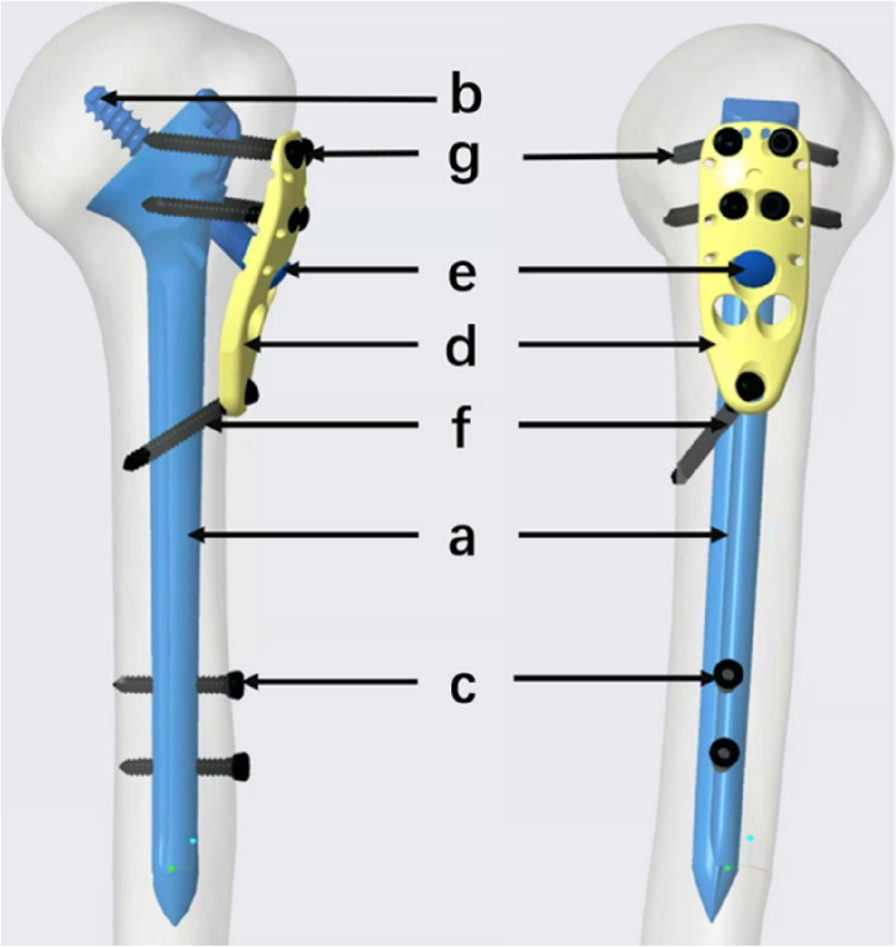
The composition of the ISNPs. **a**: intramedullary support nail (ISN), **b**: cannulated lag screw, **c**: interlocking screw of the ISN, **d**: locking plate, **e**: compression screw, **f**: distal locking screw of the plate, **g**: proximal locking screw of the plate. The foregoing alphabetical order indicates the order of application during surgery

The ISNPs is made of a titanium alloy through the expertise of Tianjin Walkman Biomaterial Co., Ltd. It predominantly comprises a long intramedullary support nail (ISN), a mini locking plate, and corresponding screws. The ISN is designed according to the geometry of the proximal humeral medullary cavity in older adults. The locking plate is contoured to the anatomy of the lateral aspect of the proximal humerus, with one compression screw hole, seven locking screw holes, and additional eyelets for fixing tuberosity sutures. A cannulated lag screw and compression screw junctional complex was designed for dynamic fixation of the humeral head fragment (Fig. 1). Tailored auxiliary tools are also equipped to ensure the smooth passage of all screws.

### Study population

The study was conducted in accordance with the Declaration of Helsinki and was approved by the Medical Ethics Committee of the Chinese PLA General Hospital (number S2020-011–01). Informed consent was obtained from all patients. This study was a retrospective cohort study. Between September 2019 and December 2020, patients diagnosed with 3- or 4-part PHF according to classification [18] who received operation in our hospital were eligible for analysis. All these patients exclusively received locking plate (LP) fixation or ISNPs fixation. The inclusion criteria were as follows: (1) completion of ≥ 12 months’ follow-up and (2) age ≥ 60 years at the time of the fracture. The exclusion criteria were as follows: (1) a history of shoulder surgery or chronic nonunion, (2) pathological or open fractures, and (3) serious nervous or vascular-injury complications. Finally, a total of 46 patients were included in the study. These patients were divided into two groups according to the treatment method, including 18 cases in ISNPs group and 28 cases in LP group. The operations were exclusively performed by two of our authors who were experienced with both devices in treating PHFs.

### Surgical technique

**Fig. 2:**
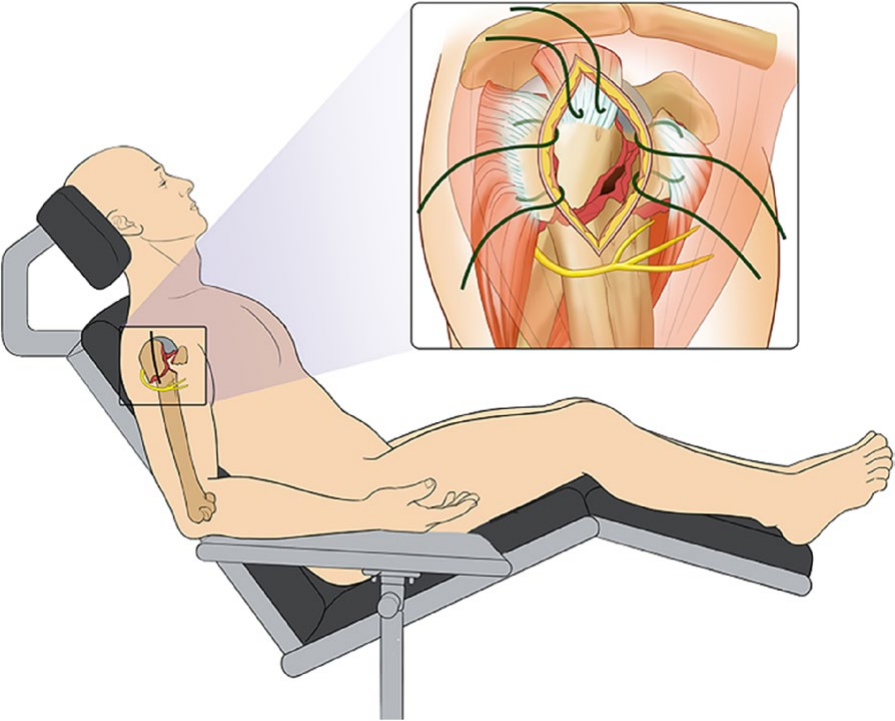
Position and surgical approach in both groups

Patients in both groups received general anesthesia and were placed in the beach-chair position. A minimally invasive deltoid splitting approach was employed. The incision commenced 1 cm posterior to the anterior acromion corner and was approximately 5–6 cm long, pointing to the lateral epicondyle. After splitting the underlying deltoid muscle and clearing the adhesive tissue, the fracture was exposed. Three #2 Ethibond® (DePuy) sutures were made in an even distribution within the tendinous insertions of the supraspinatus, infraspinatus, and subscapularis (Fig. 2).

**Fig. 3:**
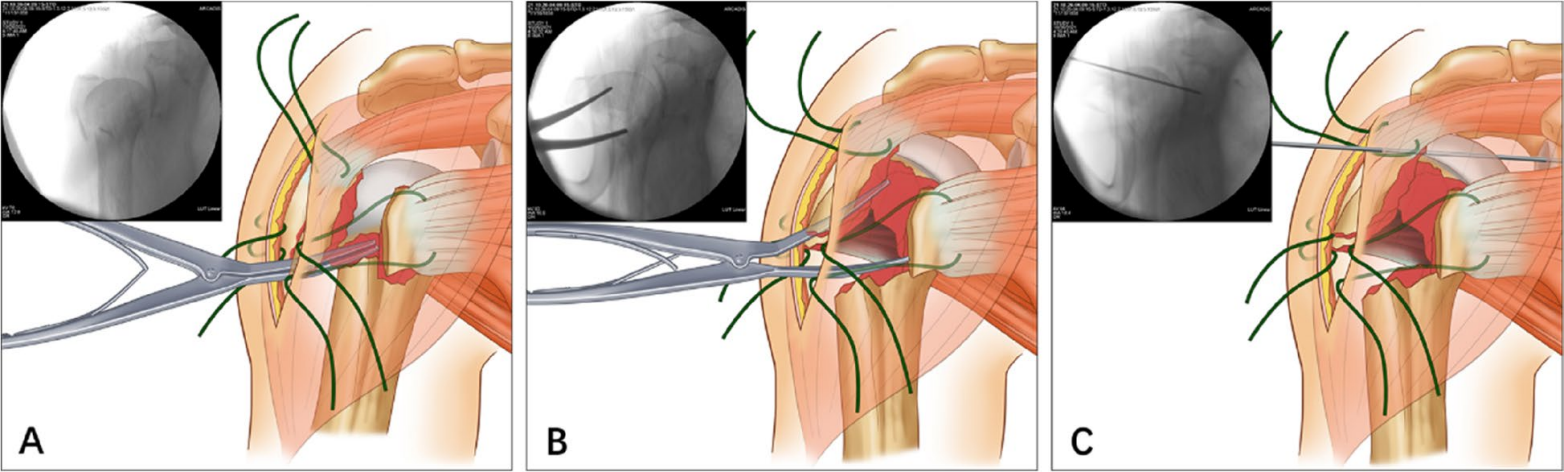
Method of humeral head fragment reduction in the ISNPs group. **A** Insertion of a laminar spreader into the medullary canal through the lateral fracture line. **B** Reduction of the humeral head by opening the spreader. **C** Provisional fixation of the humeral head to the scapular glenoid using a Kirschner wire

In the ISNs group, after thorough fracture-site exposure, a laminar spreader was inserted into the medullary canal through the lateral fracture line of the greater tuberosity (Fig. 3 A). Under fluoroscopic monitoring, the displacement of the humeral head fragment was preliminarily reduced by opening the spreader at an appropriate position with appropriate strength (Fig. 3 B). Subsequently, a K-wire was used to provisionally fix the humeral head to the scapular glenoid (Fig. 3 C).

**Fig. 4:**
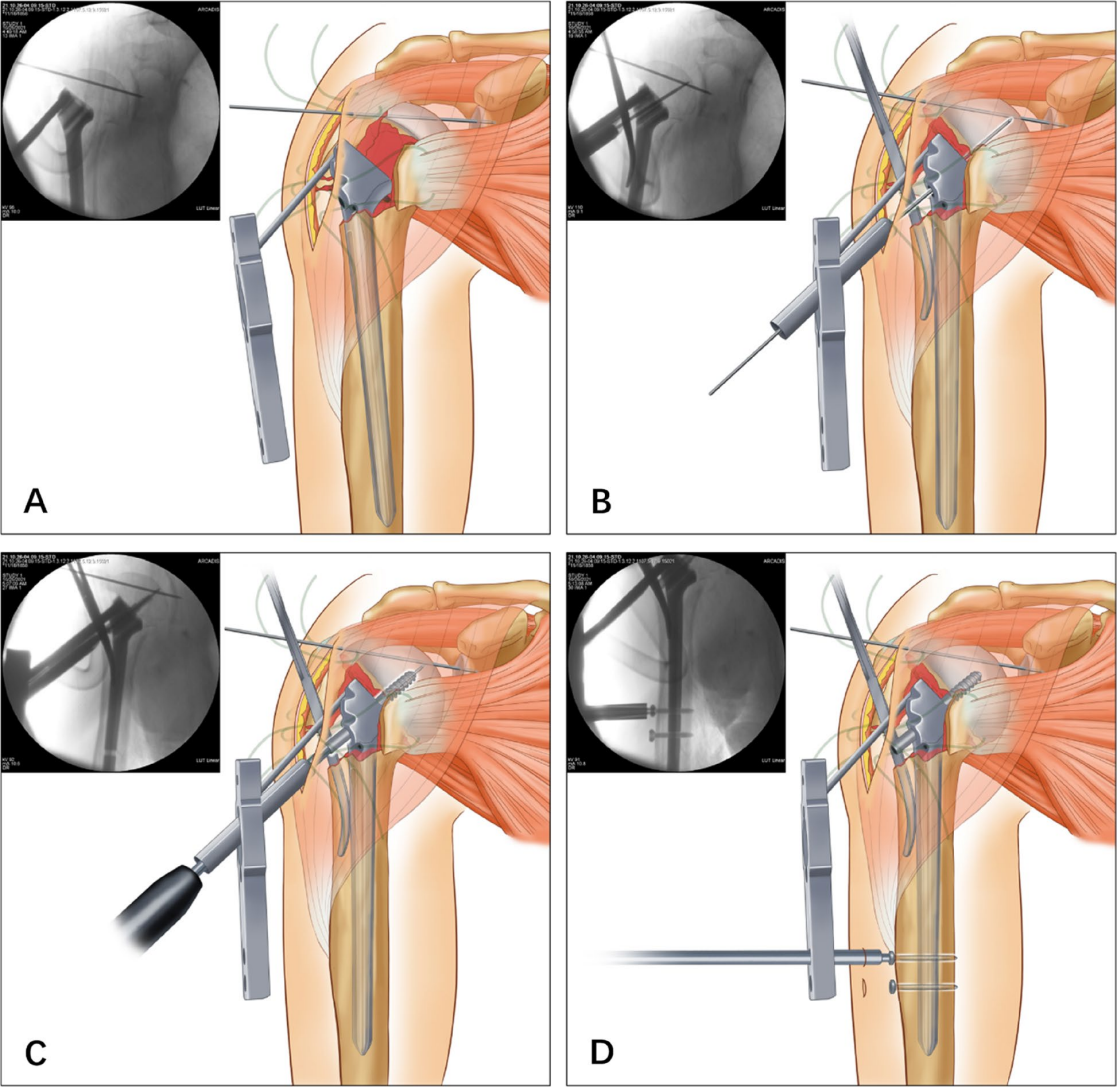
Steps of ISN fixation. **A** Insertion of the ISN into the medullary canal through the lateral fracture window. **B** Insertion of a hemostatic forceps between the ISN and lateral shaft to centralize the ISN as well as subsequent drilling of a guide pin towards the center of the humeral head. **C** Screwing of the cannulated lag screw along the guide pin. **D** Percutaneous screwing of the ISN interlocking screws

The ISN was subsequently inserted into the medullary canal through the lateral fracture window until the ISN platform approached the medial calcar of the humerus (Fig. 4 A). For enhanced reduction, hemostatic forceps were inserted between the ISN and lateral shaft to ensure that the ISN located in the medullary cavity’s center. Thereafter, a guide pin was drilled into the ISN’s sleeve, aiming at the center of the humeral head (Fig. 4 B). After length measurement, a cannulated lag screw was screwed along the guide pin, with the tip approximately 5–10 mm away from the articular surface and the base approximately 5 mm away from the lateral cortex (Fig. 4 C). After satisfactory reduction was confirmed by fluoroscopic images, the ISN’s two distal interlocking screws were placed percutaneously through the sleeves (Fig. 4 D).

**Fig. 5:**
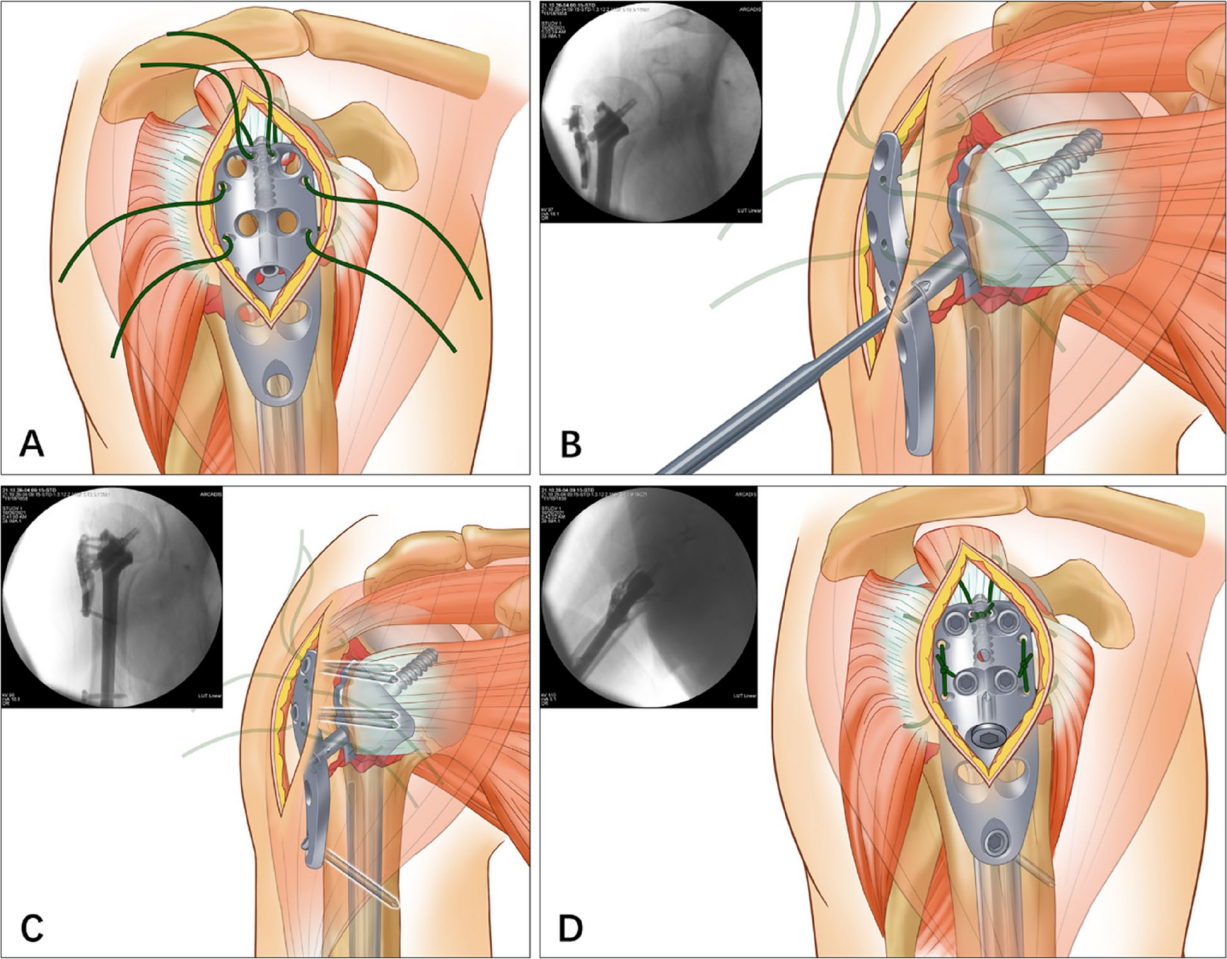
Steps of locking plate fixation. **A** Placement of the locking plate laterally to contain the greater tuberosity, while passing the sutures through the corresponding eyelets. **B** Connecting of the plate and lag screw with a compression screw and provision of proper compression. **C** Screwing in of the distal and proximal locking screws of the plate. **D** Fastening of tension sutures of the rotator cuff to the plate

Solid intramedullary support was obtained after ISN fixation. The greater and minor tuberosity fragments were subsequently reduced by dragging the sutures on the rotator cuff or clamping the fracture fragments. Thereafter, the locking plate was placed laterally to contain the comminuted greater tuberosity, and the sutures were passed through the plate’s corresponding eyelets (Fig. 5 A). The plate and lag screw were subsequently connected with a compression screw, and appropriate compression was obtained by tightening the compression screw (Fig. 5 B). The plate’s locking screws were subsequently screwed in, typically including one distal screw and four proximal screws (Fig. 5 C). In this step, special attention should be focused on preventing axillary nerve injury. After satisfactory screw positioning was confirmed by obtaining fluoroscopic images in multiple planes, the rotator cuff’s tension sutures were fastened to the plate (Fig. 5 D).

In the LP group, the displaced humeral head fragment was predominantly reduced using an elevator or K-wires for joystick techniques. Tuberosity fragments were manipulated using K-wires and sutures. After the fractures were reduced and provisionally fixed with K-wires, a longer 5-hole locking plate (Synthes, Switzerland) was selected to safely insert the distal screws. Careful examination was performed to prevent axillary-nerve entrapment. At least five screws were fixed to the humeral head, and screw distribution was monitored using fluoroscopic images. A second distal incision was subsequently made to allow for distal plate fixation.

In both groups, after careful irrigation, a negative pressure drainage tube was inserted in the wound, followed by layer closure.

### Postoperative rehabilitation

The postoperative rehabilitation program was similar in both groups. For the first 4 weeks, the operated arm was rest in a thoraco-brachial sling, while allowing for passive mobilization and pendulum exercises. Then active assisted gentle range of motion was applied from 5 to 8 weeks. Strengthening and weight bearing were permitted after 8 weeks when radiological signs of bone healing were present.

### Data collection

Patient demographic data were collected in both groups. The operative time, blood loss, and complications were recorded. Subjective outcome ratings included “excellent,” “good,” “fair,” and “poor.” Functional outcomes were assessed at the final follow-up using the Constant and disability of the arm, shoulder, and hand (DASH) scores [19, 20].

Pre- and postoperative radiographs of anterior–posterior and outlet views were reviewed. The deltoid tuberosity index (DTI) was used to evaluate local bone quality [21]. The postoperative imaging follow-up times were 4, 8, and 12 weeks as well as 6 and 12 months. Fracture healing, humeral head necrosis, screw perforation, screw loosening, and internal fixation failure were comprehensively evaluated at each time point. Fracture-reduction quality was determined using head-shaft displacement and head-shaft alignment [22]. A head-shaft displacement > 5 mm or head-shaft alignment < 110° or > 150° was considered malreduction. All measurements were made using a picture archiving and communication system (see supplementary material 1, which demonstrates the methods of measuring head-shaft displacement, head-shaft alignment, and DTI). Two of our authors collected the clinical data and did the measurement together. During the measurement, if they have disagreements on the location of anatomical landmarks, the senior doctor will make the final decision.

### Statistical analysis

Statistical analysis was conducted using SPSS 23.0 (IBM, Armonk, NY, USA). Continuous variables, presented as the mean and standard deviation, were compared using Student’s t-test to detect group differences. The groups’ qualitative data were compared using the χ2 test. Statistical significance was set at *P* < 0.05.

## Results

The demographics of the ISNPs and LP groups were comparable, and there were no significant differences in age, sex, DTI, Neer-fracture type, and follow-up time between the two groups (Table 1).

**Table 1:** Demographic details of the two groups

Variable	ISNPs group (*n* = 18)	LP group (*n* = 28)	*P*-value
Mean age (range)	70.56 ± 8.28 (60–88)	72.18 ± 8.04 (60–89)	0.512
Male	3	8	0.356
DTI	1.37 ± 0.11	1.35 ± 0.09	0.513
Neer classification			0.280
3-part	8	17	
4-part	10	11	
Follow-up (months)	14.22 ± 1.78	14.32 ± 3.06	0.901

### Clinical results

The clinical results are summarized in Table 2. There were no significant differences in intraoperative bleeding and operation time between the two groups (P = 0.635 and *P* = 0.275, respectively). Regarding reduction quality, a significant difference was found in the percentage of malreduced cases between the two groups (*P* = 0.013). In the functional score analysis, significant differences were found in both Constant score (*P* = 0.041) and DASH score (*P* = 0.009) between the two groups. In terms of the patients’ subjective rating, there were 12 excellent, 4 good and 2 fair patients in the ISPN group, while 11 excellent, 5 good, 8 fair and 2 poor patients in the LP group. Significant difference was found in the percentage of “excellent” and “good” between the ISNPs and LP groups (88.9% *vs.* 60.7%, *P* = 0.038).

**Table 2:** Clinical outcomes of the two groups at 1-year follow up

Variable	ISNP group (*n* = 18)	LP group (*n* = 28)	*P* value
Intraoperative bleeding (ml)	217 ± 151	234 ± 94	0.635
Operative time (min)	163 ± 49	146 ± 47	0.275
Malreduced (%)	2 (11.1)	13 (46.4)	0.013*
Constant score	73.61 ± 8.08	66.93 ± 11.75	0.041*
DASH score	14.71 ± 12.61	26.61 ± 15.36	0.009*
“Excellent” & “good” (%)	16(88.9)	17 (60.7)	0.038*
Complications (%)	2 (11.1)	9 (32.1)	0.103

^*^p < 0.05 compared with the LP group

### Complications

During the study period, no infection, nerve injury, DVT, or nonunion occurred in either group. The main complications were humeral head necrosis in 3 cases (one case in ISNPs group and 2 cases in LP groups), screw perforation in 6 cases (all in the LP group), and cerebral infarction in 2 cases (one case in each of the ISNPs and LP groups). Humeral head necrosis in the ISNPs group exhibited no obvious clinical symptoms, and revision surgery was unnecessary at that moment (18 months after surgery). The 2 cases of humeral head necrosis in the LP group were combined with screw perforation and obvious functional disability and were managed with arthroplasty. As regards the 6 cases of screw perforation in the LP group, 3 exhibited obvious symptom and had the perforated screws removed. The other 3 patients were satisfied with the current situation and refused further treatment. The 2 patients with cerebral infarction had mild symptoms and finally recovered well. In terms of the percentage of major complications, no significant difference was found between the two groups (11.1% in the ISNPs group *vs.* 32.1% in the LP group, *P* = 0.103).

### A typical case in the ISNPs group

**Fig. 6:**
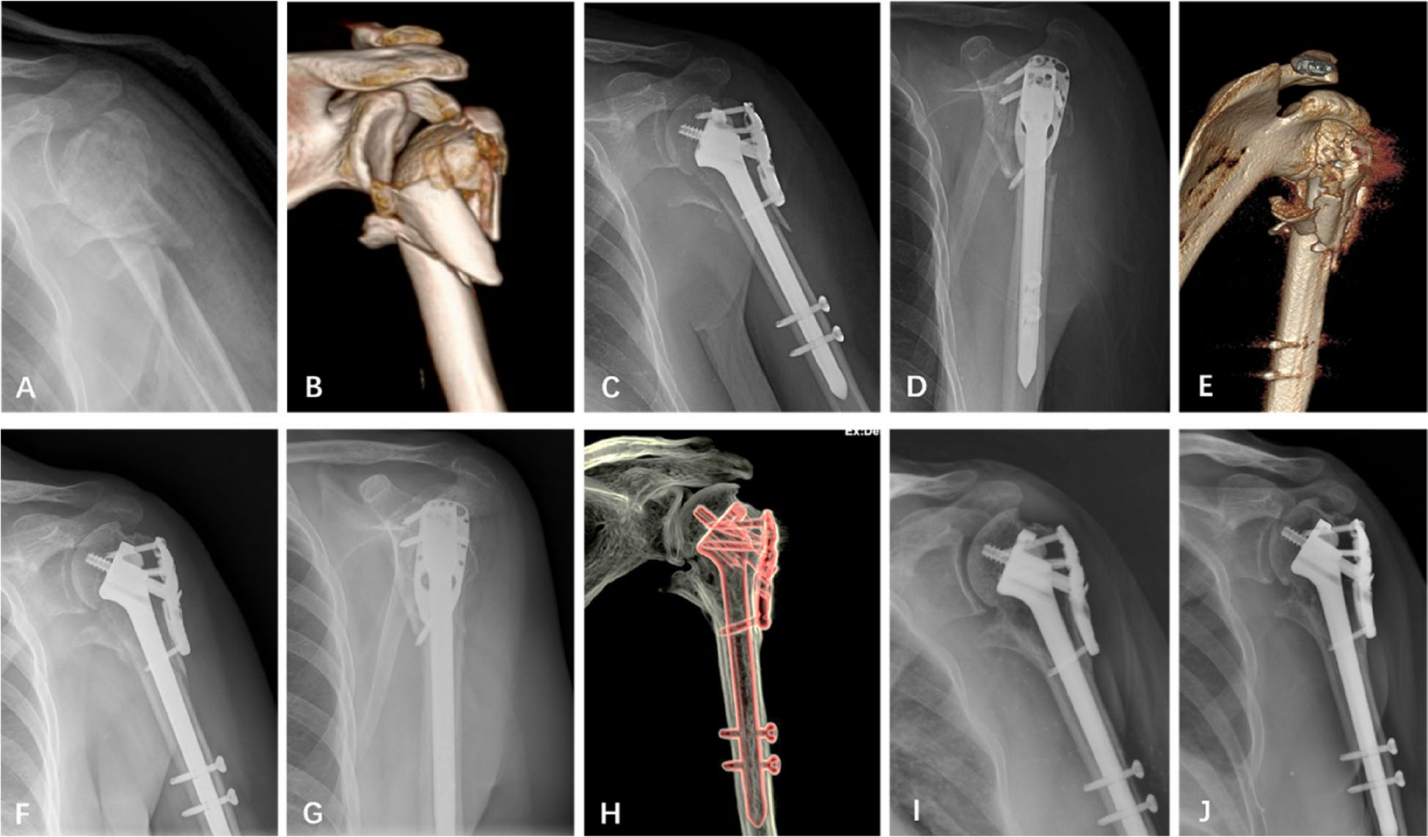
Preoperative anterior–posterior (AP) radiograph (**A**) and three-dimensional computed tomography (3D CT) reconstruction (**B**) of a shoulder revealing a 4-part PHF with comminuted medial cortex. Postoperative AP (**C)** and outlet view (**D)** radiographs and 3D CT reconstruction (**E)** of the shoulder revealing favorable head-shaft alignment, despite the medial cortex remaining obviously displaced. Three months postoperatively, the AP (**F)** and outlet view (**G)** radiographs and 3D CT reconstruction (**H)** of the shoulder revealed fracture healing without obvious reduction loss. Six (**I)** and 12 (**J)** months postoperatively, AP radiographs of the shoulder revealed firm healing of the displaced medial cortex to the humeral shaft and remolding over time

A 61-year-old woman sustained a 4-part PHF with a comminuted medial cortex due to a low-energy fall (Fig. 6 A, B). The ISNPs fixation technique was used during the operation. The postoperative radiographs revealed a well-reduced head-shaft alignment; however, the medial cortex remained significantly displaced (Fig. 6 C, D). Postoperative three-dimensional computed tomography (3D CT) reconstruction confirmed complete loss of medial shaft support (Fig. 6 E). Three months postoperatively, the fracture healed without obvious reduction loss (Fig. 6 F,G,H). At 6 and 12 months’ follow-up, the displaced medial cortex was found to have healed firmly to the humeral shaft and remolded over time (Fig. 6I,J).

**Fig. 7:**
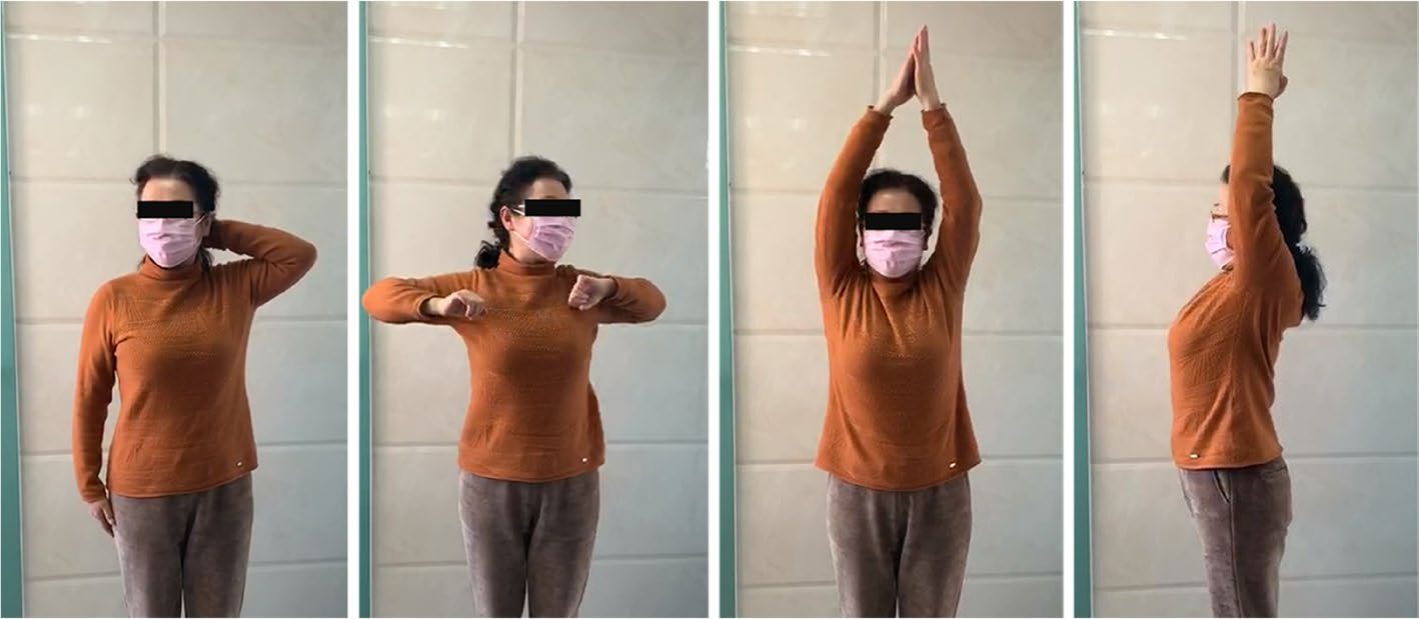
Functional status of the typical patient at 12-months’ follow-up

This patient well followed the postoperative rehabilitation program and achieved favorable functional results during the follow-up period (Fig. 7).

## Discussion

As the most common surgical treatment for PHF, internal fixation potentially provides initial stability, enables early functional rehabilitation, and improves clinical results. However, the high incidence of complications has always questioned its superiority, especially for 3- or 4- part PHF in older patients [1, 7, 8]. Some studies have concluded that surgical treatment does not yield better clinical results than non-surgical treatment for displaced PHF [23, 24]. Therefore, if the functional defects or complications resulting from non-surgical treatment are unacceptable, it is necessary to explore more effective techniques to treat these complex PHF with osteoporosis.

Many previous studies have found that locking plate fixation with fibular strut allograft augmentation potentially achieves better clinical results in treating complex PHF with osteoporosis than locking plate alone [12–15]. However, the source limitations and inherent defects of allograft transplantation have limited its clinical application. Recently, some studies explored new instruments or techniques to enhance the stability of the internal fixation of PHF. For example, Acklin et al. developed a new gliding plate-screw system to enable dynamic fixation of PHF. However, the biomechanical study did not show significant differences comparing with the conventional locking plate [25]. Clinical studies have used titanium mesh or intramedullary cages to enhance the stability of plate fixation, and encouraging results have been achieved at 1-year follow-up [26, 27]. However, fundamental technological innovations developed specifically for complex PHF with osteoporosis have rarely been reported.

Therefore, based on our previous anatomical and biomechanical studies [16, 17], we developed the ISNPs for the fixation of complex PHF with osteoporosis. As an essential part of the ISNPs, the ISN possesses the anatomical structure of the medullary cavity and potentially provides sufficient stability and intramedullary support. The locking plate, which is predominantly used for tuberosity protection, is designed to be as small as possible to facilitate a minimally invasive surgical approach. The design of the lag screw and compression screw junctional complex is modeled on the dynamic hip screw mechanism, which can realize dynamic fixation of the humeral head fragment. Based on the above characteristics, the ISNPs not only provides stable intramedullary support but also carries the advantages of locking plates and dynamic screw fixation.

Anatomical reduction of the medial column and tuberosities as well as metaphyseal buttressing have been recognized as key elements of load-sharing fixation [2, 28]. In this study, the quality of reduction in the ISNPs group was superior to that in the LP group, potentially indicating favorable clinical outcomes [4, 22]. Osteoporosis and comminution are generally considered adverse factors for fracture reduction and fixation. However, to some extent, they are conducive to ISNPs application because when the tuberosities are comminuted and the medullary cavity is spacious, ISN insertion is easier. In most cases, once the ISN is inserted in the appropriate position, reductions in both head-shaft displacement and head-shaft alignment are realized automatically. Medial comminution has been shown to significantly reduce the stability of PHF fixation structures [29]. However, the most prominent advantage of the ISNPs is its strong support for the medial column. As shown in the typical case, the comminuted medial cortex was not reduced during surgery. However, the ISNPs could still provide effective medial support, until the medial column was completely healed.

According to the clinical results, the ISNPs group showed significant advantages over the LP group in the reduction quality, functional score analysis and subjective outcome ratings. Furthermore, although not statistically significant, the complication rate of ISNPs group was much lower than those in the LP group. These findings indicate that ISNPs fixation may be a promising technique for the treatment of complex PHF in older adults.

Despite the encouraging clinical results, there is still room for improvement in the ISNPs design, according to our clinical experience. For example, multiple ISNPs models should be provided to cater to different body sizes or fracture types. Although it has not yet occurred, in the case of deep infection or other complications that need to remove the whole system of ISNPs, it would be quite difficult. The main limitations of this study are its observational nature, relatively small sample size and short follow-up time. The choice of treatment was mainly based on the preferences of patients, rather than being randomized. Further randomized controlled studies with larger sample sizes are necessary.

## Conclusion

The ISNPs was developed to combine the advantages of intramedullary support, locking plate, and dynamic fixation into a hybrid fixation system. The clinical study verified the feasibility of applying it through a minimally invasive approach to fix 3- or 4-part PHF in older patients, and it achieved favorable clinical results at 1-year follow-up. These results suggest that the application of a hybrid internal fixation system to the treatment of complex PHF with osteoporosis is a promising endeavor.

## Supplementary Information

**Additional file 1:** **Supplementary material 1.** Measurements on the picture archiving and communication system (AnyPACS 2.0; Medcare Digital Engineering Co., Ltd, Qingdao). The head-shaft alignment angle (A) was formed according to the intersection angle between the humerus shaft axis and the line perpendicular to the anatomical neck. Head-shaft displacement (B) was based on the linear relationship between the medial edge of the head part and medial edge of the shaft fracture. The DTI (C) was calculated using the ratio between the outer cortical and inner endosteal diameters (a/b) at the level directly proximal to the deltoid tuberosity.

## Data Availability

All data generated or analyzed during this study are included in this published article.
