# Supplementary material for: Technique and clinical results of a new intramedullary support nail and plate system for fixation of 3- or 4- part proximal humeral fractures in older adults
Source: BMC Musculoskelet Disord. 2022 Nov 30;23:1033. doi: 10.1186/s12891-022-05998-z (PMC9710141; doi:10.1186/s12891-022-05998-z)
Supplement: Supplementary file 1 — Additional file 1: Supplementary material 1. Measurements on the picture archiving and communication system (AnyPACS 2.0; Medcare Digital Engineering Co., Ltd, Qingdao). The head-shaft alignment angle (A) was formed according to the intersection angle between the humerus shaft axis and the line perpendicular to the anatomical neck. Head-shaft displacement (B) was based on the linear relationship between the medial edge of the head part and medial edge of the shaft fracture. The DTI (C) was calculated using the ratio between the outer cortical and inner endosteal diameters (a/b) at the level directly proximal to the deltoid tuberosity. [file 12891_2022_5998_MOESM1_ESM.docx]

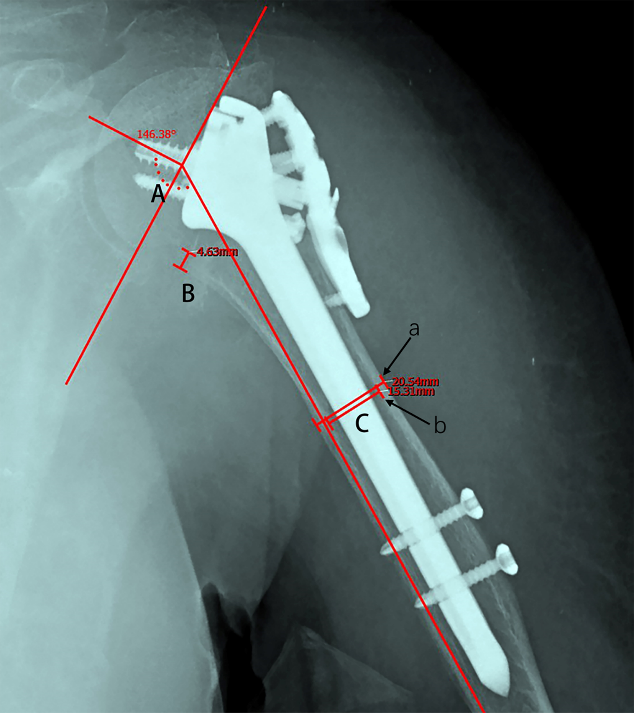


**Supplementary material 1** Measurements on the picture archiving and communication system (AnyPACS 2.0; Medcare Digital Engineering Co., Ltd, Qingdao). The head-shaft alignment angle (A) was formed according to the intersection angle between the humerus shaft axis and the line perpendicular to the anatomical neck. Head-shaft displacement (B) was based on the linear relationship between the medial edge of the head part and medial edge of the shaft fracture. The DTI (C) was calculated using the ratio between the outer cortical and inner endosteal diameters (a/b) at the level directly proximal to the deltoid tuberosity.
